# Project T-SHARP: study protocol for a multi-site randomized controlled trial of tele-harm reduction for people with HIV who inject drugs

**DOI:** 10.1186/s13063-023-07074-w

**Published:** 2023-02-07

**Authors:** Hansel E. Tookes, Asa Oxner, David P. Serota, Elizabeth Alonso, Lisa R. Metsch, Daniel J. Feaster, Jessica Ucha, Edward Suarez, David W. Forrest, Kathryn McCollister, Allan Rodriguez, Michael A. Kolber, Teresa A. Chueng, Sheryl Zayas, Bernice McCoy, Kyle Sutherland, Chetwyn Archer, Tyler S. Bartholomew

**Affiliations:** 1 Division of Infectious Diseases, Department of Medicine, University of Miami Miller School of Medicine, Miami, FL USA; 2 Department of Internal Medicine, University of South Florida Morsani College of Medicine, Tampa, FL USA; 3 Division of Health Services Research and Policy, Department of Public Health Sciences, University of Miami Miller School of Medicine, Miami, FL USA; 4 Department of Sociomedical Sciences, Mailman School of Public Health, Columbia University, New York, New York USA; 5 Biostatistics Division, Department of Public Health Sciences, University of Miami Miller School of Medicine, Miami, FL USA; 6 Department of Psychiatry, University of Miami Miller School of Medicine, Miami, Florida, USA; 7 Department of Anthropology, University of Miami, Miami, FL USA; 8 Care Resource, Fort Lauderdale, Florida, USA

**Keywords:** HIV, People who inject drugs, Harm reduction, Telehealth, Syringe services program

## Abstract

**Background:**

The resurgence of HIV outbreaks and rising prevalence among people who inject drugs (PWID) remain exigent obstacles to Ending the HIV Epidemic in the USA. Adapting a low threshold, comprehensive treatment model for PWID with HIV can leverage syringe services programs (SSPs) to increase availability and accessibility of antiretrovirals (ART), medications for opioid use disorder (MOUD), and hepatitis C cure. We developed *Tele-Harm Reduction*, a telehealth-enhanced, harm reduction intervention delivered within an SSP venue.

**Methods:**

The T-SHARP trial is an open-label, multi-site, randomized controlled superiority trial with two parallel treatment arms. Participants (*n*=240) recruited from SSPs in Miami, Ft. Lauderdale, and Tampa, Florida, who are PWID with uncontrolled HIV (i.e., HIV RNA>200) will be randomized to *Tele-Harm Reduction* or off-site linkage to HIV care. The primary objective is to compare the efficacy of *Tele-Harm Reduction* for initiation of ART at SSPs vs. off-site linkage to an HIV clinic with respect to viral suppression across follow-up (suppression at 3, 6, and 12 months post randomization). Participants with HIV RNA<200 copies/ml will be considered virally suppressed. The primary trial outcome is time-averaged HIV viral suppression (HIV RNA <200 copies/ml) over 3-, 6-, and 12-month follow-up. Secondary outcomes include initiation of MOUD measured by urine drug screen and HCV cure, defined as achieving 12-week sustained virologic response (negative HCV RNA at 12 weeks post treatment completion). A cost-effectiveness analysis will be performed.

**Discussion:**

The T-SHARP Trial will be the first to our knowledge to test the efficacy of an innovative telehealth intervention with PWID with uncontrolled HIV delivered via an SSP to support HIV viral suppression. *Tele-Harm Reduction* is further facilitated by a peer to support adherence and bridge the digital divide. This innovative, flipped healthcare model sets aside the traditional healthcare system, reduces multi-level barriers to care, and meets PWID where they are. The T-SHARP trial is a pragmatic clinical trial that seeks to transform the way that PWID access HIV care and improve HIV clinical outcomes.

**Trial registration:**

ClinicalTrials.gov NCT05208697. Trial registry name: Tele-Harm Reduction. Registration date: January 26, 2022.

## Administrative information

**Table:** 

Title {1}	T-SHARP: Telehealth Solution for HIV and Addiction-Related Problems among People who Inject Drugs.
Trial registration {2a and 2b}.	NCT05208697.
Protocol version {3}	Protocol Version 1.0.
Funding {4}	NIDA DP2DA053720.
Author details {5a}	Division of Infectious Diseases, Department of Medicine, University of Miami Miller School of Medicine, Miami, Florida, USA. Department of Internal Medicine, University of South Florida Morsani College of Medicine, Tampa, Florida, USA. Division of Health Services Research and Policy, Department of Public Health Sciences, University of Miami Miller School of Medicine, Miami, Florida, USA Department of Sociomedical Sciences, Mailman School of Public Health, Columbia University, New York, New York, USA Biostatistics Division, Department of Public Health Sciences, University of Miami Miller School of Medicine, Miami Florida, USA Department of Psychiatry, University of Miami Miller School of Medicine, Miami, Florida, USA Department of Anthropology, University of Miami, Miami, Florida, USA Care Resource, Fort Lauderdale, Florida, USA
Name and contact information for the trial sponsor {5b}	Dr. Minnjuan Flourney Floyd, Program Official, National Institute on Drug Abuse Minnjuan.flournoyfloyd@nih.gov.
Role of sponsor {5c}	The content is solely the responsibility of the authors and does not necessarily represent the official views of the National Institutes of Health.

## Introduction

### Background and rationale {6a}

The recent decade’s resurgence of HIV outbreaks and rising prevalence among people who inject drugs (PWID) remain exigent obstacles to Ending the HIV Epidemic (EHE) [1–5]. PWID account for a substantial portion of new HIV infections in the USA, which increased 8.7% between 2015 and 2019 [2, 6, 7]. Historically, PWID experience disproportionately higher HIV burden associated with sexual and injection behavior, including sexual coercion and sharing of drug preparation equipment [8, 9]. More than half (55.2%) of individuals engaging in injection drug use have hepatitis C virus (HCV) [10–12]. Their suboptimal health outcomes are often perpetuated by structural harms, including limited resources, the ongoing overdose crisis, difficulty accessing treatment for substance use, and unstable housing [4, 10, 13]. A recent drop-in HIV clinic intervention among a cohort of housing unstable individuals with substance use disorder (SUD) and uncontrolled HIV in San Francisco reported only 44% viral suppression at 12 months [14]. Furthermore, PWID living with HIV face intersecting stigmas that affect adherence to care in the traditional healthcare setting and to antiretroviral therapy (ART), and subsequently viral suppression [15–17].

The COVID-19 pandemic further complicated access to care for PWID, ushering in a sharp rise in opioid overdoses [18]. In-person harm reduction strategies were difficult to implement during the first year of the pandemic, with 43% of syringe services programs (SSPs) initially reporting a decrease in the availability of treatment services, and a quarter having to shut down entirely [19, 20]. In post-COVID adaptation of SSP programming, HCV cure, medications for opioid use disorder (MOUD), and HIV viral suppression in the high incidence group of PWID are urgently needed.

Highlighted as a cornerstone intervention in the four pillars of the EHE initiative, SSPs are low-barrier community programs uniquely positioned to provide cost-effective harm reduction services while protecting against infectious disease transmission [21–23]. Wraparound health promotion services at SSPs include the provision of sterile injection equipment, naloxone provision, wound care for treatment of skin and soft tissue infections, and HIV and hepatitis C screening, and many offer pre-exposure prophylaxis (PrEP), ART, and MOUD [23, 24].

Onsite medication treatment at SSPs has improved pharmacologic adherence and subsequently health outcomes for PWID [25, 26]. Variations of onsite HCV treatment colocated at SSPs may bundle testing, phlebotomy, and medication distribution. These studies report high rates of uptake and more than 89% of patients achieving sustained virologic response [26, 27]. One low-barrier onsite buprenorphine treatment program reported one-third of SSP participants continuing MOUD for 180 days or more [25]. Treatment retention among PWID with HIV on MOUD is significant compared to those not on MOUD, as one non-SSP systematic review and meta-analysis found that MOUD was associated with a 69% increase in ART initiation, 2-fold increase in adherence, and 45% increase in odds of HIV viral suppression [28].

Telehealth harnessed during the COVID-19 pandemic improved the treatment gap for people with HIV who use drugs, decreasing barriers of transportation and expanding outreach to remote communities, especially those experiencing homelessness [29, 30]. For people with HIV, telehealth helped providers overcome many of the factors described as barriers to care, including fear of judgement in face-to-face consultations and concerns of privacy and confidentiality [31]. Emergency regulatory changes waived the in-person clinician visit for buprenorphine prescribing, allowing for low-barrier telehealth-enhanced harm reduction interventions to be delivered to sites often frequented by PWID, including SSPs [29]. One SSP-centered telehealth model [32] offered successful co-treatment for HIV, substance use disorder, and HCV with similar rates of SVR as treatment colocated on site at an SSP [26]. However, currently no trials exist that focused on achieving viral suppression with PWID with HIV by employing a harm reduction approach in the setting of an SSP.

Adapting a low threshold, comprehensive treatment/harm reduction model for PWID with HIV can leverage trusted SSPs to increase availability and accessibility of ART, MOUD, and HCV cure. We developed *Tele-Harm Reduction* (THR), a telehealth-enhanced, harm reduction intervention delivered by an SSP that already has partnerships with the PWID community including those experiencing homelessness [33, 34]. The overall goal of this project is to compare the efficacy of the *THR* intervention in achieving HIV viral suppression among PWID with uncontrolled HIV infection in accessing services at an SSP compared to current standard of care (i.e., off-site linkage to HIV care).

### Objectives {7}

The primary objective is to compare the efficacy of *THR* for initiation of ART at SSPs vs. off-site linkage to an HIV clinic with respect to viral suppression across follow-up (suppression at 3, 6, and 12 months post randomization). Participants with HIV RNA<200 copies/ml will be considered virally suppressed.

The primary hypothesis is that *THR* will be superior to off-site linkage in achieving HIV RNA<200 copies/ml measured by the proportion of participants virally suppressed across follow-up (time-averaged viral suppression).

The secondary objectives are to measure differences between study arms in MOUD initiation and HCV cure. Participants in the *THR* arm will have enhanced access to medical and mental health clinician visits via a telehealth platform and remote phlebotomy in addition to medication management (e.g., storage in pill lockers at SSP, weekly delivery) and appointment reminders. Participants assigned to the off-site linkage arm will be linked to a traditional Ryan White clinic by SSP staff as is currently the standard of care. MOUD initiation is measured by buprenorphine, naltrexone, or methadone on urine drug screen (UDS). HCV cure (i.e., HCV RNA undetectable) is defined as sustained virologic response after completing direct acting antivirals (DAA), i.e., SVR12. Mediating effects of the intervention on injection risk behaviors (i.e., syringe reuse and syringe sharing) will also be assessed.

For the economic analysis, *THR* is expected to have a higher cost but be more effective in increasing uptake of ART, MOUD, and DAA, as well as HIV viral suppression, MOUD retention, and HCV cure among PWID participants. Incremental cost-effectiveness ratios (ICERs) will be calculated to report the additional cost per unit of desired outcome (e.g., increased ART and MOUD initiation) in the *THR* vs. off-site linkage to HIV care arms.

### Trial design {8}

The T-SHARP trial is a non-blinded, multi-site, randomized controlled superiority trial with two parallel treatment arms. Participants who are PWID with uncontrolled HIV (i.e., HIV RNA>200) will be randomized to *THR* or off-site linkage to HIV care in a 1:1 block randomization. A cost-effectiveness analysis will be performed. The study protocol (version 1.0) follows the Standard Protocol Items: Recommendations for Interventional Trials (SPIRIT) Statement.

## Methods: participants, interventions and outcomes

### Study setting {9}

Miami, Ft. Lauderdale, and Tampa are optimal cities for studying *THR* in the context of SSPs. IDEA Miami and IDEA Tampa are both housed within academic medical centers (University of Miami, University of South Florida) in partnership with safety-net hospitals (Jackson Health System, Tampa General Hospital). In consultation with our study sponsor, a third site was funded in September 2022 for the T-SHARP trial, also in an EHE jurisdiction, at the Federally Qualified Health Center-based SSP, the SPOT located in Ft. Lauderdale, Florida.

### Eligibility criteria {10}

Eligibility criteria include the following: (1) age 18 or older; (2) able to speak English; (3) enrolled in IDEA Miami, IDEA Tampa, or the SPOT SSPs; (4) injection drug use in past 12 months by self-report; (5) willing and able to sign informed consent, provide locator information and medical records release; (6) testing reactive for HIV by rapid test; and (7) HIV RNA>200 copies/ml as determined by on-site labs or abstracted medical records (result within 3 months of randomization date). Of note, pregnant people are eligible for enrollment in this trial.

Exclusion criteria include the following: (1) testing HIV negative via rapid test; (2) receipt of THR intervention in the past 6 months; (3) inability to provide informed consent; (4) planning to leave the area within 12 months; (5) principal or site investigator discretion; (6) currently in prison or jail; and (7) enrollment in NIDA Clinical Trials Network CTN 121. Investigator discretion could include serious medical, psychiatric, or co-occurring substance use disorder that acutely requires a higher or different level of care including the following: (1) disabling or terminal medical illness (e.g., decompensated heart failure, cirrhosis, or end-stage liver disease); (2) severe, untreated, or inadequately treated psychiatric condition (e.g., active psychosis); (3) in need of medical detox for severe alcohol, benzodiazepine, or other depressant or sedative hypnotic use; (4) suicidal intent or plan; or (5) homicidal ideation.

### Who will take informed consent? {26a}

Study staff including the study coordinator, research assistant, and peer counselor will recruit participants and obtain informed consent. Screening informed consent is obtained at the fixed site or mobile SSP after a participant has screened reactive for HIV. The main informed consent is obtained after a participant is determined to be eligible for the study.

### Additional consent provisions for collection and use of participant data and biological specimens {26b}

We will request consent for review of participants’ medical records, and for the collection of blood samples to assess HIV viral load. In detail, study participants will be administered full informed consent in English. Pre-screening consists of confirming that the participant is an SSP client, has a new anonymous reactive HIV test, or is known to have HIV and is out of care or non-adherent. Pre-screening is documented in the pre-screening log. If preliminary criteria are met, participants will complete the screening informed consent process including medical record release and then phlebotomy for HIV RNA. Medical record abstraction may be used to determine immediate eligibility. Consent for screening will be obtained by study staff using an IRB-approved written consent form. For those who meet eligibility criteria and would like to enter the study, informed consent will be obtained by study staff using an IRB-approved written consent form. All participants will be required to sign the medical record release form. All participants will be required to provide locator information including phone number, email, social media accounts (e.g., Facebook, Instagram), and friend/family contact information. Participants will receive a copy of all consent documents.

No additional studies are planned at this time.

## Interventions

### Explanation for the choice of comparators {6b}

The T-SHARP study is proposed as an efficacy trial because the study team has already demonstrated the feasibility and acceptability of rapid ART initiation via telehealth in PWID [17]. THR will be based out of SSPs due to their central role as a “home-base” in the community of PWID. Three sites are proposed in Florida where SSPs are currently operating—IDEA Miami, IDEA Tampa, and the SPOT Ft. Lauderdale. All study participants will be registered clients of their respective SSPs. The study will be a randomized 2-arm trial with participants randomized to either *THR* with enhanced telehealth services for ART, MOUD, and DAA that can be accessed where the patients are (e.g., SSP, mobile unit, outreach at homeless encampment, or patient home) or to off-site linkage with support from an SSP linkage specialist. Recruitment will take place at the Miami, Tampa, and Ft. Lauderdale SSPs and participants will be assessed at 3, 6, and 12 months following intake to measure uptake of ART, MOUD, and DAA; HCV cure for those who test HCV RNA positive; and risk behaviors (e.g., co-occurring stimulant use, syringe sharing, sexual risk). Quantitative assessments will help estimate predictors of HIV viral suppression via the *THR* intervention package. It is expected that syndemic factors [35, 36] such as unstable housing and/or food insecurity will impact HIV viral suppression across time points and that the *THR* intervention will perform better at mitigating the effects of these potential confounders.

### Intervention description {11a}

#### Tele-Harm Reduction intervention arm

Component 1 of the *THR* intervention utilizes telehealth technology facilitated by a peer harm reduction counselor to connect the participant with medical case managers and enroll patients in Ryan White/AIDS Drug Assistance Program (ADAP) or verify participant insurance, as prerequisite steps for initiation of HIV treatment. The peer harm reduction counselor (i.e., peer) is a person with lived experience with HIV, SUD, and/or a mental health disorder who works at the SSP. Technology is available on site at the SSPs via a HIPAA-compliant videoconferencing application, iPads, and headphones for privacy. Signed paperwork containing PHI is emailed via encryption to Ryan White and ADAP case managers**.**

Once enrollment documentation is completed, the peer will use a HIPAA-compliant videoconferencing platform to connect the patient to an on-demand visit with an HIV provider. Execution of this portion of the intervention requires secure internet access, disposable headphones to ensure the patient privacy, and mobility to meet the participant wherever they are located. Via videoconference, the physician will evaluate the patient and document the encounter in the electronic health record. Utilizing on-site phlebotomy at the SSP or on mobile outreach, we can obtain any indicated labs. All phlebotomy will occur in confidential settings following appropriate precautions.

Once the initial visit with the physician is completed, HIV medications are e-prescribed and sent to the pharmacy, determined by insurance eligibility. If the participant is immediately enrolled in ADAP, ART prescription will be sent to ADAP. If the participant is part of Department of Health (DOH) Rapid Test and Treat [37], the pharmacy will be one of the DOH-contracted specialty pharmacies. Third-party authorization to pick up medications on behalf of the participant and consent for storage of medication is obtained by the peer as part of the initial visit, and medications are either picked-up or delivered directly to the SSP for the participant to access. Once ART is obtained by the peer, the peer will observe the participant taking the first dose of the medication. The time to complete component 1 of the intervention is approximately 3 h.

Component 2 of the THR intervention utilizes the SSP-based peer harm reduction counselor to work with participants in identifying individual-specific barriers and facilitators to medication adherence and is outlined in the THR Intervention Manual. Since many participants have unstable housing, they are offered flexible medication management protocols that include on-site storage of HIV medications via pill lockers at the SSP. If participants are unable to physically retrieve their medications at the fixed site location, the peer will complete a medication drop, delivering medications directly to the participant where they are. During each medication distribution, the peer will work with participants to improve their self-efficacy to take their HIV medications using evidence-based techniques, such as motivational interviewing [38].

If participants have co-occurring mental health disorders, peers can use telehealth to connect the participants to the SSP’s on-site psychologist for mental health treatment. The psychologist will consult with the study clinician about any mental health diagnoses so that appropriate prescriptions can be initiated (e.g., SSRI for depression or anxiety). *THR* medical services include management of HIV, SUD, and other comorbidities. Infections such as skin and soft tissue infections will be treated with antibiotics, and any required procedural intervention will be referred to on-site wound care at the SSP. Physician visits are available to patients on-demand during most business hours and coordinated by the study team. All participants with OUD are repeatedly offered initiation of buprenorphine-naloxone or naltrexone, navigation to methadone programs, and naloxone for overdose prevention. Participants with chronic HCV infection are offered treatment with DAAs. Participants are screened for sexually transmitted infections and navigated to treatment when necessary. THR medical care will take an adaptive approach based on patient vulnerability (e.g., unstable housing, severe SUD, concurrent SUDs) as outlined in the THR Intervention Manual. The adaptive approach will, at the discretion of the study clinician, appropriately address patient needs (e.g., medications for stimulant use, medications for alcohol use disorder). If residential treatment for SUD is requested and appropriate, the peer will work with the participant and SSP staff to link to Ryan White or other treatment beds. This multidisciplinary team will assist the patient for 12 months, bringing the technology (i.e., traveling with iPad and WiFi hotspot) and *THR* to the participant where they are located. After the study period, the study participant will be offered continuation in the *THR* program.

Participants in the *THR* arm will have all intervention sessions audio recorded (i.e., physician, psychologist and peer encounters). Participants will be given the option to opt out of being audio-recorded.

#### Off-site linkage to HIV care arm

If randomized to off-site linkage to HIV care (control condition), the study staff will introduce the participant to an SSP HIV/HCV linkage specialist and discuss linkage to a traditional Ryan White clinic, Federally Qualified Health Center, or private physician office that treats HIV and HCV (based on the preferences of the study participant). The linkage specialist will activate the Test and Treat process within 7 days and follow standard of care procedures for linking to Ryan White case management and first provider appointment. DAA and MOUD would be received via the off-site clinic. This approach is outlined in the Intervention Manual and will include a warm handoff (i.e., linkage specialist will accompany participant and provide logistical support) to both case management and the clinician. Participants will not be linked to study clinicians but may be linked to other providers within a clinic where study clinicians see patients. At the end of the 12-month study period, patients randomized to off-site linkage arm will be offered entry into the *THR* program.

### Criteria for discontinuing or modifying allocated interventions {11b}

There will be no special criteria for discontinuing or modifying allocated interventions.

### Strategies to improve adherence to interventions {11c}

In order to assure fidelity to the *THR* intervention, all intervention sessions with the physician, psychologist, and peer will be audio-recorded. Participants may decline to be audio-recorded. Throughout the trial, lead team investigators will periodically review recordings to provide feedback to the *THR* interventionists (i.e., physician, psychologist, peer) to ensure fidelity to the intervention. Recordings will also be used for training purposes.

### Relevant concomitant care permitted or prohibited during the trial {11d}

Participants must agree to the possibility of receiving telehealth-enhanced access to HIV care via the SSP but that would not preclude them from engaging with other HIV/AIDS service organizations, primary care providers, or substance use treatment programs for further support.

### Provisions for post-trial care {30}

After study completion, participants in both arms will be offered the SSP-based *THR* intervention.

### Outcomes {12}

Table 1 shows the outcomes.

**Table 1:** Outcomes

Primary outcomes	Definition	Measure
Viral suppression	HIV viral load <200 copies/ml	HIV viral load <200 copies/ml time-averaged over 3, 6, and 12 months post baseline
Secondary outcomes	Definition	Measure
Initiation of MOUD	Receiving prescription and taking first dose of buprenorphine, naltrexone, or methadone	Positive UDS for buprenorphine, naltrexone, or methadone at study follow-up visit after MOUD is prescribed
HCV cure	Achieving 12-week sustained virologic response	HCV treatment initiated resulting in negative HCV RNA at 12 weeks post treatment completion
Cost-effectiveness	Cost per unit of a desired outcome (e.g., cost per viral suppression; cost per HCV cure)	Incremental cost-effectiveness ratios (ICERs) over 3, 6, and 12 months post baseline.

The primary trial outcome is HIV viral suppression. Viral suppression will be defined as HIV RNA <200 copies/ml which will be time-averaged HIV viral suppression over 3-, 6-, and 12-month follow-up. Secondary outcomes include initiation of MOUD defined as receiving a prescription and taking first dose of buprenorphine, naltrexone, or methadone. MOUD initiation will be measured by UDS positive for buprenorphine, naltrexone, or methadone at study follow-up visit at 3, 6, or 12 months. An additional secondary outcome is HCV cure, defined as achieving 12-week sustained virologic response. HCV cure will be measured by negative HCV RNA at 12 weeks post treatment completion.

### Participant timeline {13}

**Fig. 1:**
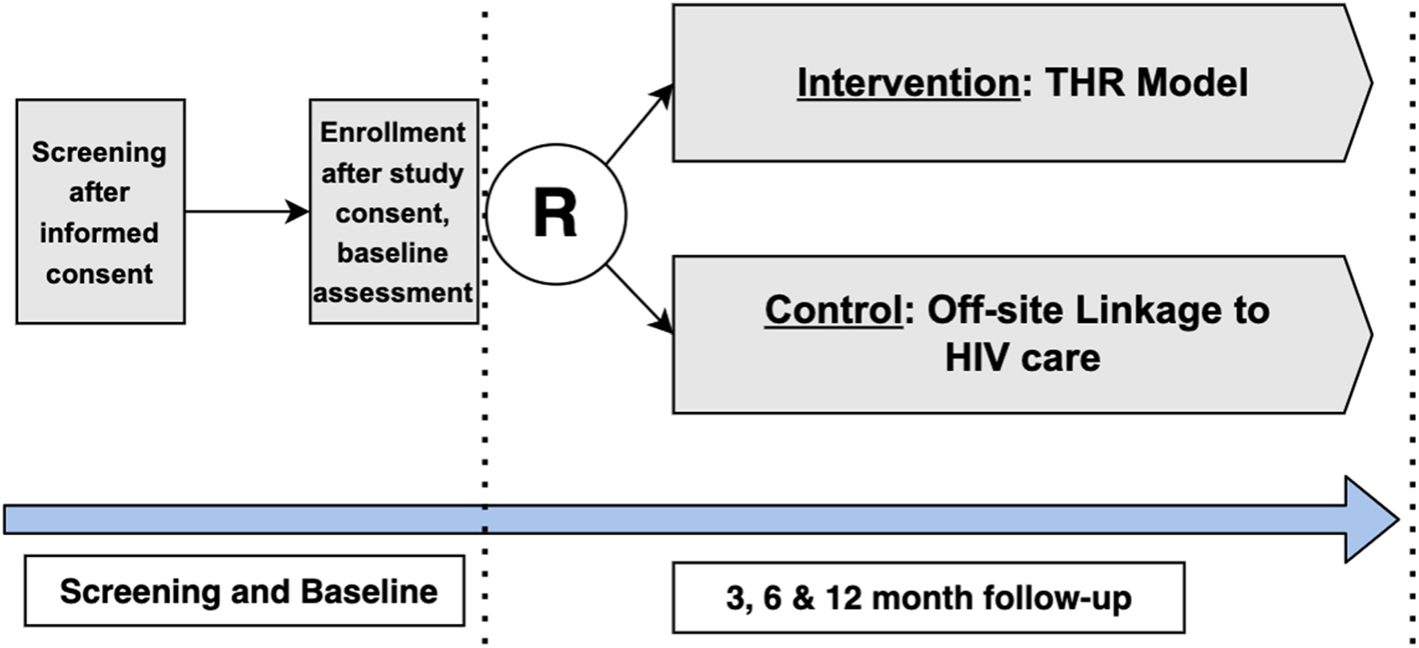
RCT study flow

Participant timeline is presented in Fig. 1.

### Sample size {14}

Assuming *α* = 0.05, power=.80, and *N*=240, we computed the minimum detectable odds ratio (*OR*) and absolute proportion difference (*pdiff*), assuming the three post-intervention time points are part of the contrast. We varied the within-subject correlation *ρ* from 0.10 to 0.80. Power for a binomial is lowest when the base-rate (control group) probability (*P*_0_) is near 50%. Minimum detectable effects are 15% absolute difference in proportions and depend on the base-rate of suppression in the standard of care (*P*_0_) and the level of autocorrelation. This size of effect falls within published cutoffs of small to medium effect sizes [39].

Power analyses were generated using the generalized estimating equations-based two-group repeated proportion module in NCSS PASS 2020 to compute minimum detectable effect sizes for the proposed primary analysis. The study will begin with 240 participants evenly assigned to the intervention and control groups (80 per city). Our primary analysis strategy will assume any participant dropping out of the study has not achieved the outcome.

### Recruitment {15}

Participants will be recruited from three SSPs by our racially and ethnically diverse staff that includes peers. Recruitment will occur by referral during routine HIV/HCV screening that is offered at the SSP every 3 months. SSP staff will refer individuals with reactive results or known HIV infection. Flyers will be posted at the SSP and on social media accounts. We will also recruit via referrals from participants in the trial, and from clinicians caring for PWID in the hospital. Interested participants will meet with the peer or other study staff for a description of study activities, opportunities, and alternatives. Interested participants will sign the screening informed consent form to determine eligibility.

It is anticipated that throughout the recruitment period (months 18–48), routine HIV screenings and active and passive referrals will facilitate identification of 300 participants with HIV. Of these, we expect 80% to be eligible for randomization. We plan to use a locator form that we have used in other NIDA Clinical Trials Network studies that includes friend and family contacts, hangout spots, and social media accounts, in addition to phone and email.

For completing the screening process including quantitative assessment and laboratory analysis, participants will receive $40. Baseline data will be collected after main study informed consent, and participants will receive $50 per laboratory assessment (0, 3, 6, 12 months) and $50 per quantitative assessment (0, 6, 12 months) in both arms. Additionally, enrolled participants may refer up to 4 individuals to the study and receive $50 each if randomized. Total possible compensation is $590.

In anticipation of recruitment challenges, the study team has been awarded an administrative supplement to add a third trial site at the SPOT in Ft. Lauderdale, Florida, a federally qualified health center-based SSP. The current IRB-approved protocol has not been updated to include 3 sites at the time of publication.

## Assignment of interventions: allocation

### Sequence generation {16a}

Participants (*n*=240) will be randomized by block randomization to *THR* or off-site linkage to HIV care. All randomizations will be stratified by gender and site (Tampa vs. Miami vs. Ft. Lauderdale). Randomization will occur with central control using the REDCap program and will be external to the three sites to assure robust and unbiased approach. Participants are randomly assigned to one of two treatment groups in a 1:1 ratio. Randomization procedure will use a permuted-block randomization scheme to ensure relative balance across time. The block size is randomly permuted with varying block sizes [4, 6, 8] to prevent study staff from guessing group assignment. Randomization tables are separated by site and stratified by biological sex.

### Concealment mechanism {16b}

Centralized randomization occurs in REDCap and group assignment is concealed until randomization.

### Implementation {16c}

The study coordinator and the research assistant will enroll eligible participants into the trial for random assignment after approval by the study principal investigator or local site principal investigator. The statistician encodes REDCap with the allocation sequence which assigns treatment condition after the eligibility REDCap form has been completed by the principal investigator.

## Assignment of interventions: blinding

### Who will be blinded {17a}

This clinical trial is unblinded. For a robust and unbiased approach, our randomization procedure is strong with central control external to the three sites. All data analysis strategies are pre-specified and the finalized data analysis plan will be confirmed prior to data lock. The data analysis will not be blinded since TSB and DJF are study investigators and performing the analysis. TSB and DJF will, however, be blinded to treatment assignment in conducting their analysis until results are finalized.

### Procedure for unblinding if needed {17b}

N/A. Study is open-label, unblinded.

## Data collection and management

### Plans for assessment and collection of outcomes {18a}

Data collection is planned at screening, baseline, and at 3-, 6- and 12-month follow-up assessments. Quantitative behavioral questionnaires will be available in English and completed in REDCap under multi-factor authentication at UM. Measures will be included from NIDA’s Data Harmonization projects, particularly NIDA’s Seek Test Treat and Retain for Vulnerable Populations data consortium. Table 1 describes the battery of quantitative assessments planned.

Laboratory assessments will include indicated laboratories in PWID. At screening, these will include HIV RNA to determine eligibility as well as T cell subsets, HCV RNA, HBsAg, HBsAb, anti-HAV, RPR, and 3-site gonorrhea and chlamydia screening. Additional laboratory assessments occur at 0, 3, 6, and 12 months. HIV RNA is measured to confirm primary outcome at 3, 6, and 12 months. HCV RNA and UDS are measured to confirm secondary outcome measures in both arms.

For the cost and cost-effectiveness analyses, we will perform an economic evaluation of *THR* versus off-site linkage to HIV care. We will look at the cost to achieve a unit of effectiveness as defined by the main study and, to the extent possible, will look at this from different perspectives (healthcare, societal). For cost data, we will create custom resource trackers based on the DATCAP method [40]. DATCAP was designed as a program-level costing instrument for standard modalities of SUD treatment.

**Table:**
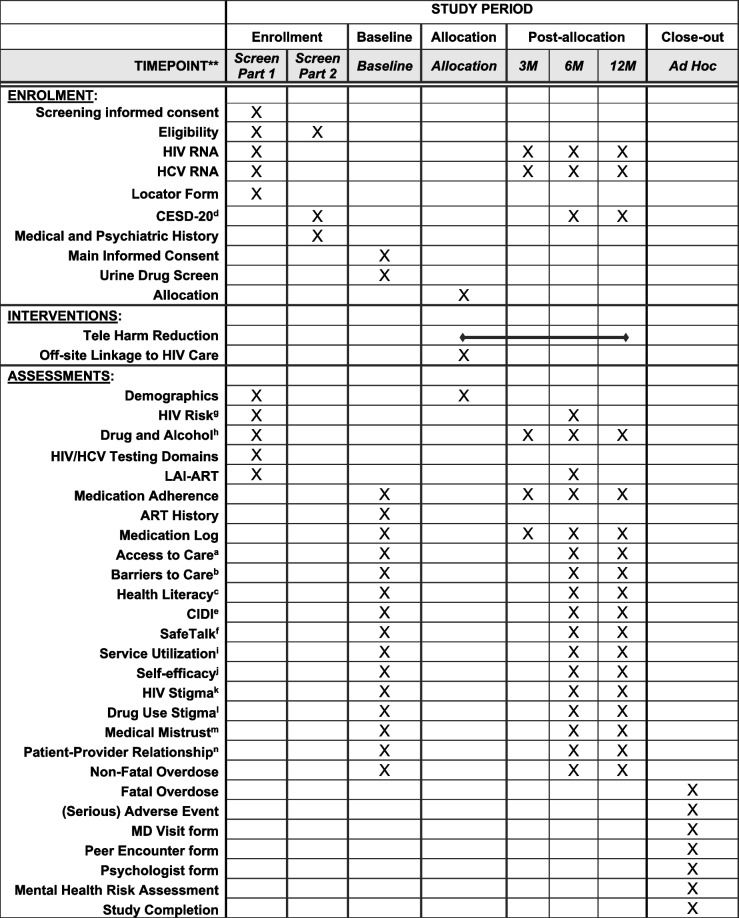


Potential moderators: ^a^Access to Care Subscale [41]; ^b^Kalichman’s Barriers to Medical Care [42]; ^c^Health Literacy Screener [43]; ^d^Center for Epidemiologic Studies Depression Scale (CES-D) [44]; ^e^Composite International Diagnostic Interview (CIDI) [45]; ^f^SafeTalk Visual Analog Scale Aid [46]; ^g^HIV Risk Measure; ^h^Drug and Alcohol Measure [47]; ^i^Service Utilization Measure [48]

Potential mediators: ^j^Medication Self-Efficacy Scale [49];^k^Berger HIV Stigma Scale [50]; ^l^Illicit Drug Use Stigma Scale [51]; ^m^Group-based medical mistrust [52]; ^n^Physician-Patient Relationship Scales [53]

### Plans to promote participant retention and complete follow-up {18b}

Participants will be recruited from IDEA Tampa, IDEA Miami, and the SPOT Ft. Lauderdale SSPs. Due to the trust that the SSPs have within the community of PWID, we anticipate that utilization of the harm reduction approach of meeting potential participants where they are will support ongoing recruitment and retention into the trial due to the potential benefits of participation (telehealth access to a physician, psychologist, counselor). Study staff are experienced and trusted in the community of PWID, and our selection of peers as harm reduction counselors will facilitate recruitment and retention. Since all participants will be recruited via an SSP, the routine exchange of syringes and street outreach will ensure ongoing contact with all participants in the trial. Participants typically visit the program weekly and this ongoing distribution of harm reduction supplies will allow the study team to retain participants in the trial. Our experience with NIDA CTN clinical trials, street outreach, and trust in the community should facilitate retention. We also plan to use a locator form that we have used in other CTN studies that includes friend and family contacts, hangout spots, and social media accounts, in addition to phone and email. Additionally, IDEA Miami has a three-person community engagement team that regularly conducts street outreach, home visits, medication deliveries, appointment reminders, Ryan White enrollment, housing assistance, and linkage to treatment for SUD. This team is being replicated in Tampa and Ft. Lauderdale. The team has close ties with local homeless shelters, treatment programs, law enforcement, Federally Qualified Health Centers, health department, and community mental health centers that will facilitate retention. The affiliation of all sites with the safety-net health system (Jackson Health System in Miami, Tampa General Hospital, and Broward Health/Memorial Healthcare System) will aid retention.

### Data management {19}

A study-specific data management protocol and standard operating procedures will guide the study team throughout the trial. All data management activities will utilize REDCap and forms will be tested prior to implementation. REDCap is secure, HIPAA-compliant, web-based and has real-time validation rules at the time of entry. Data will be reviewed for completeness on an ongoing basis and reviewed for quality control. The lead statistician will provide ongoing oversight of data management throughout the study and supervise Dr. Bartholomew in generating reports for quality control and data analysis. A study-specific data dictionary will be developed by all members of the research team. Data management reports will be made to the DSMB. After database lock, cleaned, de-identified data files will be produced for analysis.

### Confidentiality {27}

In terms of confidentiality, all data will be kept confidential, electronically password protected, and available only to authorized staff. The passwords will change periodically and accessible only to specified study staff. Participants’ data will be identified by an ID number only in the REDCap database. The link between names and ID numbers will be kept separately under password protection that only the site PIs can access*.* All participants will be advised that they may decline to answer any study question. These procedures will be implemented to provide study participants with the assurance of confidentiality around sensitive and personal information relating to their mental health, sexual and substance use behaviors, and HIV status. All study personnel working on the project will be trained in human subject research, good clinical practice, and the importance of strictly respecting participants’ rights to confidentiality.

### Plans for collection, laboratory evaluation, and storage of biological specimens for genetic or molecular analysis in this trial/future use {33}

The primary and secondary outcomes will be measured with HIV RNA, HCV RNA, and buprenorphine, naltrexone, or methadone on UDS. These laboratories are completed as routine blood and urine lab tests, sent to a commercial laboratory, and destroyed according to their procedures.

## Statistical methods

### Statistical methods for primary and secondary outcomes {20a}

#### Preliminary analyses and missing data

Frequency tables for all variables and measures of central tendency and variability for continuous variables will characterize the sample and be stratified by randomization group (i.e., intervention versus control). As recommended by CONSORT guidelines (http://www.consort-statement.org/), we will not do statistical tests comparing randomized groups at baseline. Our primary analyses will assume participants who drop out and have no available medical records have not achieved our outcome (e.g., HIV viral suppression) and therefore missing data will not affect the primary analyses. In additional analyses, we will relax this assumption and treat dropout as missing data. In addition, for our moderator (and mediator) hypotheses, there may be some missing data on self-reported syndemic factors, for example. The data analysis for this study will be generated using SAS software, Version 9.4, trademarks of SAS Institute Inc., Cary, NC, USA. The proposed analyses will be conducted using validated algorithms. All program code and results will be documented extensively and archived to enable future review, transparency, and result reproducibility.

#### Hypothesis 1

*THR* will be superior to off-site linkage to HIV care in achieving HIV RNA<200 copies/ml measured by the proportion of participants virally suppressed across the follow-up assessments.

#### Primary inferential analyses to address primary aim (Hypothesis 1)

We hypothesize that, for those randomized to the *THR* arm, the odds of our primary outcome, HIV viral suppression, will be higher than for those randomized to off-site linkage to HIV care (control) arm (Hypothesis 1). Generalized estimating equations (GEE) [54] will be used to perform the proposed primary analysis, which is a planned time-averaged comparison of post baseline measurements of HIV viral suppression across the *THR* and control groups to test primary Hypothesis 1. Alpha will be set at 0.05 for this planned comparison. Planned post hoc comparisons will examine the difference between conditions at each of the three assessment times.

Though GEE estimates are consistent even if the correlation structure is mis-specified, GEE’s statistical efficiency improves as the working correlation structure more closely approximates the actual correlation structure, so various correlation structures suitable for the study’s design will be considered (e.g., exchangeable, autocorrelated, *m*-dependent, unstructured). The QIC statistic will be used to select the final correlation structure. Recruitment city (Miami vs. Tampa or Ft. Lauderdale) will be included in all models as required by the stratified randomized design to obtain unbiased results. Robust standard errors will be used to obtain correct inferences even if the chosen correlation structure remains slightly mis-specified. All analyses will include outlier and influential case screening via computation of GEE-based residual analysis, including leverage, DFBeta, and Cook’s D statistics. If outliers are found, results will be reported with and without outliers included.

#### Sex as a biological variable

We will examine the primary hypothesis and the two secondary hypotheses (see below) with respect to difference by sex. In particular, the primary analysis method will be used and a sex by treatment interaction in the time-averaged odds of viral suppression will be added to the model to test whether there are differential treatment effects by sex. With the expected 80 or 1/3 of the sample being women, there is reasonable power to uncover a difference.

#### Data analysis plan and statistical procedures to address secondary hypotheses

For preliminary analyses and missing data, the secondary analyses will use the same preliminary analysis and missing data strategy as Hypothesis 1. Hypothesis 2 is that *THR* will be superior to off-site linkage to HIV care in achieving (1) initiation of MOUD and (2) retention in MOUD as evidenced by positive UDS for MOUD. Hypothesis 3 is that *THR* will be superior to off-site referral and off-site linkage to HIV care in achieving (1) initiation of medication (DAA) for HCV Cure and (2) HCV cure as evidenced by undetectable HCV RNA at 12 weeks post HCV treatment (SVR12).

Hypotheses 2a and 3a will be tested using a logistic regression model where the dependent measure is equal to zero if the individual does not initiate the medication prior to the end of the study (month 12) and is equal to 1 if the individual does initiate the medication prior to the end of the study. We will test whether *THR* relative to the control group increases the odds of initiating the respective medication. Note that for Hypothesis 2a the sample is all participants, whereas, for 3a, it is only those eligible for HCV treatment, i.e., who have a positive HCV viral load at baseline.

Hypotheses 2b will be tested as will be Hypothesis 1, with GEE as a time-averaged comparison of post-initiation of MOUD measurements of presence of MOUD by UDS between those randomized to *THR* and the control condition. To maintain this hypothesis test as an intention to treat analyses, in the initial test, all participants will be included. Those who have not initiated will be coded as not retained. In a planned post hoc comparison, the sample will be reduced to only those who have initiated to examine whether there is a difference in rates of retention conditional on initiation.

Hypothesis 3b will be tested as will be H2a and H3a using logistic regression. Initial intent-to-treat analyses will compare the odds of achieving HCV cure in the 12 months post randomization within the *THR* arm relative to the control arm within the full sample that was HCV positive at baseline. Planned post hoc comparisons will repeat this analysis within the sample that initiated HCV treatment to examine whether there were significant differences conditional on initiation.

The alpha will be set at .05 for each these secondary hypotheses (2a, 2b, 3a, and 3b). In each model, recruitment site (Miami vs. Tampa or Ft. Lauderdale) will be included in these analyses as required by the stratified randomized design to obtain unbiased results. To maximize rigor, appropriate diagnostics including outlier and influential case screening will be examined for each model. If outliers are found, results will be reported with and without outliers included.

#### Sex as a biological variable

We will examine each of secondary hypotheses (2a, 2b, 3a, and 3b) with respect to difference by sex. In particular, the respective analysis method will be used and a sex by treatment interaction to test whether there are differential treatment effects by sex.

### Interim analyses {21b}

If study recruitment fails to meet quotas and the original target sample size appears unlikely to be achieved, investigators will propose an updated target sample size and conduct a futility analysis. Conditional power will be calculated based on the treatment effect size observed in the current data and “information fraction” consistent with the updated target sample size. This analysis indicates the likelihood of finding a significant effect if trends in the current data continue and the updated sample size target is met. If conditional power under the updated sample size fails to meet a pre-specified threshold of 0.5, the stopping rule will be considered satisfied. If, on the other hand, conditional power is high, the trial is likely to meet its primary outcome even with the reduced sample size. The DSMB will use this information to guide its recommendation to continue or discontinue the trial.

### Methods for additional analyses (e.g., subgroup analyses) {20b}

Data analysis plan and statistical procedures to address mediation and moderation.

#### Moderation analyses

*THR* will buffer the impact of syndemic factors on hypothesized outcomes. The GEE model will be used to examine the relationship between syndemic factors and the hypothesized outcomes (HIV viral suppression, engagement in MOUD, and HCV treatment). Then an interaction between the syndemic factors and treatment assignment will test whether the impact of syndemic factors were lessened in *Tele-Harm Reduction*.

#### Mediation analyses

The impact of *THR* will be mediated through increased engagement and retention in substance use treatment.

To maximize rigor, these analyses will not be performed with classical multiple regression-based mediation techniques such as the Baron and Kenny causal steps approach [55]. Instead, mediation analyses will be conducted using structural equation modeling (SEM) and bootstrapped tests of significance of the product of coefficients (a*b, where *a* is the path coefficient from the intervention to mediator, and *b* is the path coefficient from mediator to outcome). We will use M*plus* [56] to perform mediation analyses because it unites SEM with causal inference-based mediation methods in the same analysis platform. Alpha will be set at .05 for all hypothesis tests in these exploratory analyses.

#### Cost and cost-effectiveness analysis

Using a micro-costing approach, detailed information on the costs of each strategy (*THR* and off-site linkage to HIV care) will be collected using tailored version of the standardized costing survey, the Drug Abuse Treatment Cost Analysis Program (DATCAP) [40]. The DATCAP is flexible in that it organizes resources across standard categories: personnel, consultants and contractors, buildings and facilities, equipment, supplies, donated or subsidized resources, and miscellaneous (e.g., licensing fees, administrative/overhead). It electronically generates key economic cost summary statistics using data on intervention engagement and patient case flow: total annual program cost, average annual cost per patient; average cost (per participant) per treatment episode.

### Methods in analysis to handle protocol non-adherence and any statistical methods to handle missing data {20c}

We will address any incomplete data with multiple imputation (MI) [57], which makes the relatively mild assumption that incomplete data arise from a conditionally random (MAR) mechanism [54]. Auxiliary variables will be included to help meet the MAR assumption.

### Plans to give access to the full protocol, participant-level data, and statistical code {31c}

In terms of our data sharing plan, we will provide de-identified data to interested investigators 1 year after publishing the primary outcome paper. After obtaining IRB approvals for planned analyses, and any needed data sharing plans, de-identified data will be sent.

## Oversight and monitoring

### Composition of the coordinating center and trial steering committee {5d}

For this three site proposed project, we have developed an organizational system for the study with well-coordinated data management. The data monitoring and management procedures will be established to maintain active, clear communication at each site and between sites. Specifically, Drs. Tookes, Bartholomew, Feaster, Alonso, Metsch, Oxner, and Serota will hold weekly zoom meetings with site study staff to direct study activities. This meeting schedule will be maintained throughout all years of the project. These meetings will provide general training and address consistency of procedures as well as problems and challenges. Topics will include issues of recruitment and retention, data collection/management/analysis, budget, and protocol fidelity. Drs. Tookes, Oxner, Serota, and Suarez will supervise and train harm reduction counselors in the *THR* intervention at both sites. Dr. Bartholomew will work with Dr. Feaster to generate regular quality control reports for the sites and make real-time corrections.

The *THR* manual was developed by Drs. Tookes, Bartholomew, Metsch, Oxner, Serota, and Suarez in conjunction with harm reduction counselors. Regular zoom meetings to support harm reduction counselors in the delivery of the intervention will occur. Drs. Tookes, Oxner, Serota, and Suarez will assess the extent to which study intervention was implemented in a manner that is maximally consistent with the intent of the intervention manual though periodic fidelity monitoring of intervention session audio recordings.

Data forms will undergo a rigorous systematic editing process prior to entry into the REDCap database. Dr. Bartholomew will routinely evaluate the data and discuss any problems with study staff and investigators at the weekly team meetings. Data management formal reports on record status across the three following domains will be employed: entered, verified, and edited. These reports will be evaluated once monthly during team meeting. To help ensure data protection, backup copies will be automatically generated by our computer systems. Data collected from study assessments and questionnaires will be entered directly into REDCap. Confidentiality is assured as participants will be identified on all study materials only by participant number, visit number, and date of visit. By recording the study data in this manner, the information can be considered “de-identified,” and therefore, compliant with the Standards for Privacy of Individually Identifiable Health Information (“Privacy Rule”) of the Health Insurance Portability Act of 1996 (“HIPAA”).

### Composition of the data monitoring committee, its role and reporting structure {21a}

To fulfill its mission of ensuring the safety and integrity of the study, it is necessary that the Data Safety Monitoring Board (DSMB) be comprised of members who possess a high degree of competence and experience, as well as the ability to function independently of all other parties involved in the study. The DSMB members will function free of the career and financial interests of its members and DSMB consists of three members with experience in conducting clinical intervention research on HIV treatment, expertise in biostatistics, and a thorough knowledge of clinical trial ethics and human subject protection issues. The DSMB chair is Dr. Paula Lum. Dr. Ricky Bluthenthal is the harm reductionist. The biostatistician is Dr. Ryan Cook. Dr. Allan Rodriguez will serve as medical monitor.

The DSMB will meet annually by zoom conference and be updated semi-annually by report. At the yearly meeting, members will review randomization data as well as adverse events. The following serious adverse events (SAEs) will be reported within 24 h: deaths, hospitalizations, fatal and non-fatal drug overdose, and psychiatric hospitalizations.

### Adverse event reporting and harms {22}

It is unlikely that participants will be at substantial risk for harm as a result of study participation. They will be prescribed ART and be monitored for potential medication side effects as part of their clinical care outside of specific study labs. Any drug-related toxicities (e.g., elevated creatinine) will be monitored by the study team. Additionally, participants may find some of the questions asked in the questionnaire to be upsetting. Study labs will be conducted routinely and may cause slight discomfort. There is always the potential risk of loss of confidentiality, but procedures are in place to prevent this potential risk. Using telehealth, it is possible that a sign or symptom could be missed. Peers will assist the physician and psychologist in physical diagnosis by taking photos of any wounds and securely emailing them to the clinicians. In-person exams are available at the SSPs. The physician will conduct a thorough review of systems.

We will enroll PWID with HIV initiating ART who will be queried for new potential adverse events at each research visit after screening informed consent. In the THR arm, peer harm reduction counselors will receive training to address medication non-adherence, sexual and injection risk taking, and substance use behaviors. The clinical psychologist will be available on-demand for any participant in either study arm experiencing severe emotional distress, suicidality, or homicidality. He will be backed up by the five study clinicians who have a rotating on-demand call schedule. Additionally, the SSPs have licensed mental health counselors on staff in the case of mental health crisis. During the behavioral assessments, the REDCap survey will automatically indicate provide an alert for a score on the CES-D that suggests that the person is experiencing severe depressive symptoms. Any necessary linkage to emergency services will be provided. With respect to blood draws, each of these procedures will be carried out phlebotomists trained to work with PWID or study physicians to minimize the accidental injury or discomfort to the participants. Participants who experience harm as a result of these procedures will receive first aid from study staff and referral to medical professional if needed.

#### Monitoring of safety data by the DSMB

Safety information will be reported to the DSMB in an unblinded manner. A statistical penalty will not be assessed for the ongoing unblinded review of safety by the committee. Unblinded data will not be released to site investigators unless necessary for safety reasons. If requested by the DSMB, other unblinded data will be reported to investigators. It is necessary for the purpose of monitoring the safety of the study that the committee review not only adverse events (AEs) and SAEs, but also other data that may reflect differences in safety between groups, including retention rates and reasons for dropout.

Expedited review by the medical monitor will occur for all events meeting the FDA definition of SAE. SAEs include any event that a study investigator or the DSMB judges to impose a significant hazard, contraindication, or side effect. The following SAEs will be reported in REDCap within 24 h: deaths, hospitalizations, fatal and non-fatal drug overdose, and psychiatric hospitalizations. For purposes of this study, all SAEs will be required to be reported to the DSMB, regardless of any judgment of their relatedness to the study, at regular meetings. If the medical monitor deems the SAE to be study related, it will be immediately reported (within 24 h) to the DSMB via email, to the IRB if the risk to subjects was previously not known (i.e., consent/protocol change necessary), and to the NIDA Program Official by email. Unfortunately, SAEs such as fatal and non-fatal overdose are expected due to the study population. All relevant information about the event and its outcome, study condition, concomitant medications, the subject’s medical history and current conditions, and laboratory data will be reported. Information will be reviewed and a determination made of whether there was any possible relevance to the study interventions. Reporting to the IRB will be completed according to guidelines. Reporting to the NIH will be made according to their respective regulations governing SAEs. SAE reporting to the DSMB will occur at the regular meetings, unless deemed significant and related to the study.

Adverse events for this study are defined as suicidal intent or plan, injection drug use-associated infection, non-injection drug use-associated infection, new onset liver failure, new onset renal failure, and complications of phlebotomy performed at the SSP. AEs will be collected for this study during the 12-month period after randomization. SAEs are categorized above. AEs will be reported in REDCap within 7 days and presented to the DSMB and IRB yearly.

At yearly intervals during the course of the study and at completion, the IRB and the DSMB will be provided with unblinded summaries of the numbers and rates of AEs by study arm. These reports will include types and severity of events. Data on individual non-serious AEs is not expected to be needed for this review.

#### Other safety-related reports

At yearly intervals throughout the course of the study, the DSMB and IRB will also receive unblinded summary reports of intervention retention and reasons for dropout by study arm.

If at any time during the course of the study, the DSMB judges that risk to subjects outweighs the potential benefits, the DSMB shall have the discretion and responsibility to recommend that the study be terminated. The IRB may request unblinded data to make this determination at any time.

Semi-annually, during the course of the study, the DSMB will receive a report on data quality and completeness and participant flow. This report will include an overview of the progress of participant intake and retention, summaries describing participant compliance with visits, and a summary of key baseline data elements to examine balances in randomization. These reports will be used by the DSMB to evaluate the capacity of the data capture and processing to support scientifically valid analyses. Reports are done graphically, similarly to the CONSORT figures.

### Frequency and plans for auditing trial conduct {23}

As a clinical trial in a harm reduction setting, the T-SHARP trial has a rigorous quality assurance plan. That plan will include periodic “interim” site monitoring visits conducted approximately every 12 weeks. The purpose of these visits is to review study documentation for accuracy and completeness, to conduct source-to-database comparisons for data quality, and to monitor compliance with the study protocol and procedures. Interim visits may be conducted on site or remotely. Site performance rates (recruitment and retention) will be reviewed on a monthly basis and feedback will be provided to sites regarding their success in meeting performance targets. The scope of the visit will include reviewing the following documentation for 100% of consented participants: (1) informed consent forms, HIPAA forms, and medical records releases; (2) eligibility forms/documentation; (3) electronic data capture-to-source verification for data points related to primary outcome, i.e., HIV viral load <200 copies/ml at 3, 6, and 12 months post baseline; (4) AE/SAE documentation; (5) protocol deviation documentation, as applicable.

The external QA monitor will provide consultation to study Lead Team on quality-related matters including recommendations for quality controls that can be built into the procedures of the study (e.g., visit checklists/progress notes and study implementation logs; guidance regarding Regulatory File set-up for Lead Team and sites; support on the evaluation of protocol deviations and determination of corrective actions; recommendations for providing regular site-level feedback on performance targets). The QA monitor will participate in the training of study site teams, delivering training on good documentation practices and how to conduct internal quality control as part of daily study activities and providing refresher training on QA-related matters to site teams (site investigators, study coordinators, peers) as needed. QA monitor will provide on site and/or remote monitoring at (1) site initiation to assess site readiness for launch; (2) periodically throughout study implementation to assess data quality and protocol compliance; and (3) at study closeout and will submit reports of monitoring findings and track all findings to resolution. Finally, the QA monitor will attend all-site calls to remain informed on study progress and milestones, as well as specific implementation challenges faced by sites and provide QA updates and announcements on site calls.

### Plans for communicating important protocol amendments to relevant parties (e.g., trial participants, ethical committees) {25}

Any amendments to the protocol or consent will be approved by the institutional review board prior to implementation. Participants will be reconsented if the consent is amended to include new risk information (changes in the anticipated risks/benefits of participation) or other information that involves significant changes to the procedures of the study.

### Dissemination plans {31a}

Community engagement is a central component of our work in harm reduction. Accordingly, we have included PWID or are in recovery during our planning of the intervention and will continue to include them throughout the conduct of the trial. The community will inform us of the acceptability of our recruitment and retention efforts and any changes that might be needed to the protocols during the startup period. PWID will lead our active community dissemination as findings from this trial emerge. The study team will stay closely connected to the community throughout the duration of the study. We will participate in community advisory board (CAB) meetings through the UM Center for AIDS Research (CFAR) CAB, and the UM Developmental Mental Health AIDS Research Center (D-ARC or CHARM Center) CAB. We will solicit input from the CABs and PWID as to how to best stay engaged with the community in terms of dissemination of findings.

We will register the trial on ClinicalTrials.gov to ensure that results are submitted according to the required policies and on time and that registration and result information remains up to date. We will register the study before the first subject is randomized and update every 12 months at minimum. Summary results will be submitted less than 1 year after the trial’s completion. Through the University of Miami login, the PI will manage the ClinicalTrials.gov study updates. We will include information about the posting of the results on ClinicalTrials.gov on the study consent.

In addition to the main outcome paper, we plan to publish multiple manuscripts from in peer-reviewed journals. We expect to have a separate cost-effectiveness analysis manuscript. We also expect to publish at least one baseline manuscript characterizing the sample and focusing on the prevalence and impact of syndemics in the population, and one manuscript detailing the intervention. We also plan to present findings at scientific conferences after the trial is completed.

## Discussion

In the wake of multiple injection drug use-associated HIV outbreaks in the US, HIV infections among PWID have increased in recent years, with 11% of new HIV infections in the PWID community [58]. Despite emergent need in this high-priority community, the T-SHARP Trial, to the best of our knowledge, will be the first to test the efficacy of an innovative telehealth intervention delivered via an SSP to support HIV viral suppression. *Tele-Harm Reduction* is further facilitated by a peer to support adherence and bridge the digital divide [59–61]. This deconstructed healthcare model sets aside the traditional healthcare system and meets PWID where they are, both mentally and physically. The novel *Tele-Harm Reduction* intervention leverages the trusted platform of the SSP and uses technology to connect PWID with their clinicians in a destigmatizing environment. It is a peer-delivered intervention that places PWID at the center and acknowledges them as the true experts in their own health.

Viral suppression in this high incidence group is urgently needed, particularly in the Southern USA, an epicenter of substance use and HIV [62]. PWID are an under-resourced, high HIV incidence community, but unfortunately, there are few evidence-based interventions to engage PWID with HIV in care and promote viral suppression, and none to our knowledge in harm reduction settings. Adapting a low threshold, comprehensive treatment model for PWID with HIV can leverage trusted SSPs to increase availability of ART, MOUD, and HCV cure. We developed a *Tele-Harm Reduction* intervention to help PWID overcome the psychosocial and structural burden aspects of the syndemics theoretical framework [35, 36]. Preliminary data suggest that SSP-based telehealth for HIV care is both feasible and acceptable and pilot data showed 78.1% (*n*=35) viral suppression at 6-month follow-up [17]. Our novel, white glove, concierge medicine approach to PWID health shows great promise as an efficacious model of care for a long-overlooked community. In pursuit of sustainability, we will conduct a cost-effectiveness analysis as part of the T-SHARP trial.

There were challenges for an early-stage investigator in planning and launching the trial in the COVID-19 pandemic as well as bringing a rigorous NIDA Clinical Trials Network-level study to a harm reduction setting, which by definition is less formal than a traditional healthcare setting. The close affiliation of the IDEA Miami SSP with the University of Miami Miller School of Medicine and IDEA Tampa with University of South Florida facilitated trial implementation while remaining authentic to a harm reduction approach [63]. Addition of a rigorous quality assurance plan to the trial ensured trial readiness. Furthermore, there were challenges related to implementation of the trial at new SSPs. Both IDEA Tampa and the future trial site at the SPOT Broward opened during the COVID-19 pandemic, adding complexities to both establishment of core harm reduction services while simultaneously readying the programs for research worthy of the National Institutes of Health. Unfortunately, growth in enrollment in all three SSPs has been slowed by the pandemic which could add recruitment challenges and thus necessitated early planning for a third trial site, funded by a NIDA administrative supplement.

The T-SHARP trial is a pragmatic clinical trial [64] that seeks to transform the way that PWID access HIV care. It is high risk, high reward, and thus appropriately funded through the DP2 Avenir Award mechanism. Future research should expand the trial for a status-neutral *THR* approach—specifically, a strengths-based, broader reach, destigmatized approach that does not identify people by their diseases (HIV, HCV, i.e., status neutral [65]), embodies the true principles of harm reduction, and meets people where they are with respect, dignity, warmth, and care to reduce the harms associated with drug use. A comprehensive, status-neutral approach could adapt the *Tele-Harm Reduction* intervention for MOUD, PrEP, HCV treatment, and beyond with transformative potential.

## Trial status

The current protocol in use is version 1 (approved September 26, 2022). The trial started recruiting October 10, 2022. The anticipated end date for recruitment is April 30, 2025.

## Data Availability

Any data required to support the protocol can be supplied on request to the PI.

## Abbreviations

ADAP: AIDS Drug Assistance Program
AE: Adverse event
ART: Antiretroviral therapy
CAB: Community Advisory Board
CES-D: Center for Epidemiologic Studies Depression Scale
CIDI: Composite International Diagnostic Interview
CTN: Clinical Trials Network
DAA: Direct acting antivirals
DOH: Department of Health
DSMB: Data Safety Monitoring Board
EHE: Ending the HIV Epidemic
GEE: Generalized Estimating Equations
HCV: Hepatitis C virus
HIPAA: Health Insurance Portability and Accountability Act
HIV: Human immunodeficiency virus
ICER: Incremental cost-effectiveness ratios
IDEA: Infectious Disease Elimination Act
LAI-ART: Long Acting Injectable Antiretroviral Therapy
MOUD: Medications for opioid use disorder
NIDA: National Institute on Drug Abuse
PrEP: Pre-exposure prophylaxis
PWID: People who inject drugs
QI: Quality improvement
SAE: Serious adverse event
SIRI: Severe injection-related infection
SSP: Syringe services program
STTR: Seek, Test, Treat, and Retain
SUD: Substance use disorder
T-SHARP: Telehealth Solution to HIV and Addiction-Related Problems
THR: Tele-Harm Reduction
UDS: Urine drug screen
UM: University of Miami
